# 8-Chloro-6-iodo-2-phenyl­chromeno[4,3-*c*]pyrazol-4(2*H*)-one *N*,*N*-dimethyl­formamide monosolvate

**DOI:** 10.1107/S1600536811022070

**Published:** 2011-06-18

**Authors:** Pradeep Lokhande, Kamal Hasanzadeh, Hamid Khaledi, Hapipah Mohd Ali

**Affiliations:** a Department of Chemistry, University of Pune, Pune 411007, India; b Department of Chemistry, University of Malaya, 50603 Kuala Lumpur, Malaysia

## Abstract

In the title compound, C_16_H_8_ClIN_2_O_2_·C_3_H_7_NO, the fused tricyclic pyrazolo­coumarin ring and the *N*-phenyl ring are almost coplanar, the dihedral angle between them being 1.86 (9)°. In the crystal, these rings stack on top of each other *via* π–π inter­actions [centroid–centroid distances = 3.489 (2), 3.637 (2), 3.505 (2) and 3.662 (2) Å], forming infinite chains along the *a* axis. The chains are connected into layers parallel to *ac* plane through I⋯O inter­actions [3.0011 (18) Å] between pairs of symmetry-related mol­ecules. The DMF solvent mol­ecules are C—H⋯O bonded to this network.

## Related literature

For related structures, see: Strakova *et al.* (2003 ▶); Kanwal *et al.* (2007 ▶). For a crystal structure (*p*-iodo­benzaldehyde) having I⋯O inter­actions, see: Britton & Young (1997) ▶. For a background to the I2/DMSO reagent, see: Lokhande *et al.* (2005 ▶).
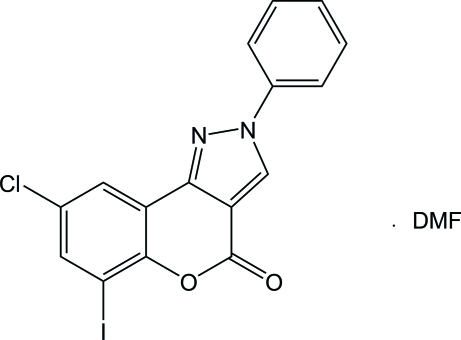


## Experimental

### 

#### Crystal data

C_16_H_8_ClIN_2_O_2_·C_3_H_7_NO
*M* *_r_* = 495.69
Triclinic, 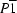

*a* = 7.7297 (5) Å
*b* = 11.5196 (2) Å
*c* = 12.0326 (3) Å
α = 118.484 (1)°
β = 99.841 (1)°
γ = 90.968 (1)°
*V* = 921.86 (7) Å^3^
*Z* = 2
Mo *K*α radiation
μ = 1.91 mm^−1^
*T* = 100 K
0.30 × 0.10 × 0.02 mm

#### Data collection

Bruker APEXII CCD diffractometer
Absorption correction: multi-scan (*SADABS*; Sheldrick, 1996 ▶) *T*_min_ = 0.598, *T*_max_ = 0.963
7205 measured reflections
3392 independent reflections
3075 reflections with *I* > 2σ(*I*)
*R*_int_ = 0.021

#### Refinement

*R*[*F* ^2^ > 2σ(*F* ^2^)] = 0.023
*wR*(*F* ^2^) = 0.052
*S* = 1.10
3392 reflections
246 parameters
H-atom parameters constrained
Δρ_max_ = 0.58 e Å^−3^
Δρ_min_ = −0.56 e Å^−3^

### 

Data collection: *APEX2* (Bruker, 2007 ▶); cell refinement: *SAINT* (Bruker, 2007 ▶); data reduction: *SAINT*; program(s) used to solve structure: *SHELXS97* (Sheldrick, 2008 ▶); program(s) used to refine structure: *SHELXL97* (Sheldrick, 2008 ▶); molecular graphics: *X-SEED* (Barbour, 2001 ▶); software used to prepare material for publication: *SHELXL97* and *publCIF* (Westrip, 2010 ▶).

## Supplementary Material

Crystal structure: contains datablock(s) I, New_Global_Publ_Block. DOI: 10.1107/S1600536811022070/go2013sup1.cif

Structure factors: contains datablock(s) I. DOI: 10.1107/S1600536811022070/go2013Isup2.hkl

Additional supplementary materials:  crystallographic information; 3D view; checkCIF report

## Figures and Tables

**Table 1:** Hydrogen-bond geometry (Å, °)

*D*—H⋯*A*	*D*—H	H⋯*A*	*D*⋯*A*	*D*—H⋯*A*
C10—H10⋯O3	0.95	2.19	3.122 (3)	167
C16—H16⋯O3	0.95	2.49	3.415 (3)	164

**Fig. 1.**
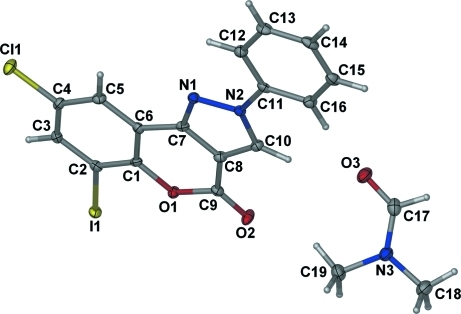
The molecular strucrure of the title compound (50% probability ellipsoids). Hydrogen atoms are drawn as spheres of arbitrary radius.

## supplementary crystallographic information

### Comment

The title compound was obtained through a cyclization/iodination reaction, using I_2_/DMSO reagent (Lokhande *et al.*, 2005). The crystal structure consists of the heterocyclic molecules, solvated by DMF molecules. The pyrazolocoumarin moiety is essentially planar (r.m.s deviation of the tricyclic ring atoms = 0.018 Å) as is in the similar structures (Strakova *et al.*, 2003; Kanwal *et al.*, 2007). The plane of the tricyclic ring is inclined slightly with respect to the *N*-phenyl ring, making an angle of 1.86 (9)°. The iodine atom and the carbonyl O atom of the symmetry related molecule at -*x*, -*y* + 2, -*z* + 1 are brought close together with I1···O2 distance of 3.0011 (18) Å which is significantly shorter than the sum of the Van der Waals radii of the relevant atoms (3.50 A°). Similar intermolecular interactions have been reported for the structure of *p*-iodobenzaldehyde (Britton & Young, 1997) with I···O distances of 3.068 (4) and 3.074 (4)Å and suggested to be an interaction between the Lewis base, –CHO, and the Lewis acid, I. The crystal packing consists of layers parallel to the *ac* plane formed by the I···O and the π-π interactions [*Cg*1···*Cg*1^i^ = 3.489 (2) Å; *Cg*1···*Cg*3^ii^ = 3.637 (2) Å; *Cg*2···*Cg*4^i^ = 3.505 (2) Å; *Cg*4···*Cg*4^i^ = 3.662 (2) Å, where *Cg*1, *Cg*2, *Cg*3 and *Cg*4 are the centroids of the rings N1/N2/C7/C8/C10, O1/C6—C9, C1—C6 and C11—C16, respectively, for *i*: -*x* + 1, -*y* + 2, -*z* + 2; *ii*: -*x*, -*y* + 2, -*z* + 2]. The DMF solvent molecules are hydrogen bonded to the layers (Table 1).

### Experimental

A solution of 5-chloro-2-hydroxyacetophenone (2.4 mmol, 0.41 g) and phenylhydrazine (2.4 mmol, 0.62 g) in methanol (40 ml), was refluxed for 2 hr to give 5-chloro-2-hydroxy acetophenone phenylhydrazone as a yellow solid (91%). To a solution of the obtained hydrazone (2 mmol, 0.52 g) in DMF (15 ml), POCl_3_ (6 mmol, 0.918 g) was added dropwise at 0 *^o^*C. After completion of the addition, the mixture was heated at 60–70 *^o^*C for 2.5–3 hr, then poured onto crushed ice and neutralized with 10% aqueous NaOH solution. The precipitate was filtered, washed with water and recrystallized from ethanol to give 3-(5-chloro-2-hydroxyphenyl)-1-phenyl-1*H*-pyrazole-4-carbaldehyde (85%). To a solution of this solid (1 mmol, 0.298 g) in DMSO (20 ml), iodine (1.2 equivalent, 0.304 g) and 4–5 drops of concentrated H_2_SO_4_ was added. The mixture was heated at 120 *^o^*C for 3 hr, then cooled to room temperature and poured into ice-cooled water. The separated solid was filtered and washed with a cold dilute sodium thiosulfate solution and recrystallized from DMF to give the colorless crystals of the title compound.

### Refinement

Hydrogen atoms were placed at calculated positions and refined as riding atoms with distances of H—C(*sp*^2^) = 0.95 and *H*—*C*(methyl) = 0.98 Å and with*U*_iso_(H) set to 1.2(1.5 for methyl)*U*_eq_(C). The most disagreeable reflections with delta(*F^2^*)/e.s.d. >10 were omitted (5 reflections).

### Crystal data

**Table:**

C_16_H_8_ClIN_2_O_2_·C_3_H_7_NO	*Z* = 2
*M**_r_* = 495.69	*F*(000) = 488
Triclinic, *P*1	*D*_x_ = 1.786 Mg m^−^^3^
Hall symbol: -P 1	Mo *K*α radiation, λ = 0.71073 Å
*a* = 7.7297 (5) Å	Cell parameters from 4487 reflections
*b* = 11.5196 (2) Å	θ = 2.7–30.3°
*c* = 12.0326 (3) Å	µ = 1.91 mm^−^^1^
α = 118.484 (1)°	*T* = 100 K
β = 99.841 (1)°	Needle, colorless
γ = 90.968 (1)°	0.30 × 0.10 × 0.02 mm
*V* = 921.86 (7) Å^3^	

### Data collection

**Table:**

Bruker APEXII CCD diffractometer	3392 independent reflections
Radiation source: fine-focus sealed tube	3075 reflections with *I* > 2σ(*I*)
graphite	*R*_int_ = 0.021
φ and ω scans	θ_max_ = 25.5°, θ_min_ = 2.0°
Absorption correction: multi-scan (*SADABS*; Sheldrick, 1996)	*h* = −9→9
*T*_min_ = 0.598, *T*_max_ = 0.963	*k* = −13→13
7205 measured reflections	*l* = −13→14

### Refinement

**Table:**

Refinement on *F*^2^	Primary atom site location: structure-invariant direct methods
Least-squares matrix: full	Secondary atom site location: difference Fourier map
*R*[*F*^2^ > 2σ(*F*^2^)] = 0.023	Hydrogen site location: inferred from neighbouring sites
*wR*(*F*^2^) = 0.052	H-atom parameters constrained
*S* = 1.10	*w* = 1/[σ^2^(*F*_o_^2^) + (0.0269*P*)^2^] where *P* = (*F*_o_^2^ + 2*F*_c_^2^)/3
3392 reflections	(Δ/σ)_max_ = 0.003
246 parameters	Δρ_max_ = 0.58 e Å^−^^3^
0 restraints	Δρ_min_ = −0.56 e Å^−^^3^

### Special details

**Table:**

Geometry. All e.s.d.'s (except the e.s.d. in the dihedral angle between two l.s. planes) are estimated using the full covariance matrix. The cell e.s.d.'s are taken into account individually in the estimation of e.s.d.'s in distances, angles and torsion angles; correlations between e.s.d.'s in cell parameters are only used when they are defined by crystal symmetry. An approximate (isotropic) treatment of cell e.s.d.'s is used for estimating e.s.d.'s involving l.s. planes.
Refinement. Refinement of *F*^2^ against ALL reflections. The weighted *R*-factor *wR* and goodness of fit *S* are based on *F*^2^, conventional *R*-factors *R* are based on *F*, with *F* set to zero for negative *F*^2^. The threshold expression of *F*^2^ > σ(*F*^2^) is used only for calculating *R*-factors(gt) *etc*. and is not relevant to the choice of reflections for refinement. *R*-factors based on *F*^2^ are statistically about twice as large as those based on *F*, and *R*- factors based on ALL data will be even larger.

### Fractional atomic coordinates and isotropic or equivalent isotropic displacement parameters (Å^2^)

**Table:**

	*x*	*y*	*z*	*U*_iso_*/*U*_eq_	
I1	−0.11384 (2)	1.169294 (17)	0.628935 (17)	0.01600 (7)	
Cl1	−0.02765 (10)	1.45032 (7)	1.17754 (7)	0.02134 (15)	
O1	0.0778 (2)	0.97585 (17)	0.70964 (18)	0.0149 (4)	
O2	0.1906 (3)	0.78881 (18)	0.60164 (18)	0.0198 (4)	
N1	0.2949 (3)	0.9833 (2)	1.0488 (2)	0.0135 (5)	
N2	0.3677 (3)	0.8654 (2)	0.9952 (2)	0.0123 (5)	
C1	0.0571 (3)	1.0858 (2)	0.8236 (2)	0.0127 (5)	
C2	−0.0335 (3)	1.1827 (3)	0.8102 (3)	0.0147 (6)	
C3	−0.0607 (3)	1.2950 (3)	0.9203 (3)	0.0146 (6)	
H3	−0.1242	1.3617	0.9128	0.017*	
C4	0.0055 (3)	1.3084 (3)	1.0406 (3)	0.0153 (6)	
C5	0.0988 (3)	1.2139 (3)	1.0556 (3)	0.0148 (6)	
H5	0.1450	1.2260	1.1393	0.018*	
C6	0.1240 (3)	1.1004 (3)	0.9457 (3)	0.0130 (5)	
C7	0.2193 (3)	0.9941 (3)	0.9470 (3)	0.0127 (5)	
C8	0.2419 (3)	0.8862 (3)	0.8304 (3)	0.0142 (5)	
C9	0.1744 (3)	0.8750 (3)	0.7064 (3)	0.0144 (6)	
C10	0.3400 (3)	0.8053 (3)	0.8657 (3)	0.0142 (5)	
H10	0.3796	0.7239	0.8098	0.017*	
C11	0.4601 (3)	0.8195 (3)	1.0783 (3)	0.0139 (5)	
C12	0.4788 (3)	0.8972 (3)	1.2102 (3)	0.0163 (6)	
H12	0.4323	0.9804	1.2456	0.020*	
C13	0.5671 (4)	0.8521 (3)	1.2909 (3)	0.0186 (6)	
H13	0.5817	0.9050	1.3820	0.022*	
C14	0.6333 (3)	0.7306 (3)	1.2387 (3)	0.0190 (6)	
H14	0.6921	0.6996	1.2939	0.023*	
C15	0.6140 (4)	0.6541 (3)	1.1063 (3)	0.0187 (6)	
H15	0.6606	0.5711	1.0709	0.022*	
C16	0.5267 (3)	0.6980 (3)	1.0246 (3)	0.0170 (6)	
H16	0.5131	0.6456	0.9336	0.020*	
O3	0.5295 (3)	0.5613 (2)	0.70310 (19)	0.0253 (5)	
N3	0.3732 (3)	0.3775 (2)	0.5253 (2)	0.0204 (5)	
C17	0.5161 (4)	0.4454 (3)	0.6173 (3)	0.0228 (7)	
H17	0.6171	0.3993	0.6162	0.027*	
C18	0.3726 (4)	0.2405 (3)	0.4280 (3)	0.0292 (7)	
H18A	0.4855	0.2090	0.4459	0.044*	
H18B	0.3559	0.2342	0.3426	0.044*	
H18C	0.2759	0.1854	0.4295	0.044*	
C19	0.2124 (4)	0.4393 (3)	0.5173 (3)	0.0274 (7)	
H19A	0.2335	0.5340	0.5816	0.041*	
H19B	0.1176	0.3961	0.5341	0.041*	
H19C	0.1779	0.4295	0.4307	0.041*	

### Atomic displacement parameters (Å^2^)

**Table:**

	*U*^11^	*U*^22^	*U*^33^	*U*^12^	*U*^13^	*U*^23^
I1	0.01706 (10)	0.01850 (10)	0.01258 (10)	0.00408 (7)	0.00108 (7)	0.00823 (8)
Cl1	0.0319 (4)	0.0175 (3)	0.0138 (3)	0.0083 (3)	0.0068 (3)	0.0061 (3)
O1	0.0175 (9)	0.0147 (9)	0.0114 (10)	0.0039 (8)	0.0018 (8)	0.0057 (8)
O2	0.0308 (11)	0.0178 (10)	0.0112 (10)	0.0104 (9)	0.0056 (9)	0.0068 (9)
N1	0.0135 (11)	0.0133 (11)	0.0135 (12)	0.0010 (9)	0.0012 (9)	0.0068 (10)
N2	0.0122 (11)	0.0120 (11)	0.0130 (12)	0.0005 (9)	0.0010 (9)	0.0069 (10)
C1	0.0121 (13)	0.0131 (13)	0.0099 (13)	−0.0015 (10)	0.0019 (11)	0.0035 (11)
C2	0.0121 (13)	0.0180 (14)	0.0150 (15)	0.0005 (11)	0.0017 (11)	0.0093 (12)
C3	0.0130 (13)	0.0156 (14)	0.0168 (15)	0.0031 (11)	0.0037 (11)	0.0090 (12)
C4	0.0169 (13)	0.0127 (13)	0.0145 (14)	0.0010 (11)	0.0051 (12)	0.0047 (12)
C5	0.0177 (14)	0.0163 (13)	0.0102 (14)	−0.0004 (11)	0.0014 (11)	0.0070 (12)
C6	0.0103 (12)	0.0152 (13)	0.0149 (14)	0.0006 (10)	0.0029 (11)	0.0084 (12)
C7	0.0131 (12)	0.0139 (13)	0.0119 (14)	−0.0030 (10)	0.0027 (11)	0.0069 (11)
C8	0.0159 (13)	0.0151 (13)	0.0120 (14)	−0.0002 (11)	0.0014 (11)	0.0074 (11)
C9	0.0132 (13)	0.0165 (14)	0.0146 (15)	0.0006 (11)	0.0009 (11)	0.0092 (12)
C10	0.0168 (13)	0.0134 (13)	0.0123 (14)	0.0002 (11)	0.0034 (11)	0.0060 (11)
C11	0.0095 (12)	0.0173 (14)	0.0177 (15)	−0.0024 (10)	−0.0011 (11)	0.0121 (12)
C12	0.0171 (14)	0.0175 (14)	0.0143 (14)	0.0007 (11)	0.0023 (12)	0.0080 (12)
C13	0.0180 (14)	0.0265 (15)	0.0121 (14)	−0.0012 (12)	−0.0003 (12)	0.0112 (13)
C14	0.0144 (13)	0.0268 (16)	0.0221 (16)	−0.0032 (12)	−0.0029 (12)	0.0191 (14)
C15	0.0155 (13)	0.0162 (14)	0.0256 (17)	−0.0015 (11)	−0.0003 (12)	0.0127 (13)
C16	0.0189 (14)	0.0159 (14)	0.0162 (15)	0.0015 (11)	0.0008 (12)	0.0088 (12)
O3	0.0324 (12)	0.0209 (11)	0.0172 (11)	0.0065 (9)	0.0037 (10)	0.0053 (10)
N3	0.0241 (13)	0.0185 (12)	0.0163 (13)	0.0046 (10)	0.0033 (11)	0.0068 (11)
C17	0.0267 (16)	0.0267 (17)	0.0217 (17)	0.0097 (13)	0.0093 (14)	0.0155 (15)
C18	0.0326 (18)	0.0234 (16)	0.0236 (18)	0.0047 (14)	0.0034 (15)	0.0059 (14)
C19	0.0230 (16)	0.0296 (17)	0.0278 (18)	0.0081 (13)	0.0046 (14)	0.0126 (15)

### Geometric parameters (Å, °)

**Table:**

I1—C2	2.092 (3)	C11—C12	1.380 (4)
Cl1—C4	1.744 (3)	C11—C16	1.384 (4)
O1—C9	1.380 (3)	C12—C13	1.395 (4)
O1—C1	1.391 (3)	C12—H12	0.9500
O2—C9	1.206 (3)	C13—C14	1.383 (4)
N1—C7	1.327 (3)	C13—H13	0.9500
N1—N2	1.375 (3)	C14—C15	1.383 (4)
N2—C10	1.344 (3)	C14—H14	0.9500
N2—C11	1.436 (3)	C15—C16	1.392 (4)
C1—C2	1.387 (4)	C15—H15	0.9500
C1—C6	1.398 (4)	C16—H16	0.9500
C2—C3	1.394 (4)	O3—C17	1.227 (3)
C3—C4	1.383 (4)	N3—C17	1.335 (4)
C3—H3	0.9500	N3—C18	1.449 (4)
C4—C5	1.381 (4)	N3—C19	1.454 (4)
C5—C6	1.394 (4)	C17—H17	0.9500
C5—H5	0.9500	C18—H18A	0.9800
C6—C7	1.445 (4)	C18—H18B	0.9800
C7—C8	1.404 (4)	C18—H18C	0.9800
C8—C10	1.383 (4)	C19—H19A	0.9800
C8—C9	1.437 (4)	C19—H19B	0.9800
C10—H10	0.9500	C19—H19C	0.9800
			
C9—O1—C1	123.4 (2)	C12—C11—N2	119.0 (2)
C7—N1—N2	103.4 (2)	C16—C11—N2	119.5 (2)
C10—N2—N1	113.3 (2)	C11—C12—C13	119.1 (3)
C10—N2—C11	127.5 (2)	C11—C12—H12	120.5
N1—N2—C11	119.2 (2)	C13—C12—H12	120.5
C2—C1—O1	116.1 (2)	C14—C13—C12	120.1 (3)
C2—C1—C6	121.0 (2)	C14—C13—H13	119.9
O1—C1—C6	122.9 (2)	C12—C13—H13	119.9
C1—C2—C3	119.2 (2)	C15—C14—C13	120.0 (3)
C1—C2—I1	121.04 (19)	C15—C14—H14	120.0
C3—C2—I1	119.61 (19)	C13—C14—H14	120.0
C4—C3—C2	119.4 (2)	C14—C15—C16	120.5 (3)
C4—C3—H3	120.3	C14—C15—H15	119.8
C2—C3—H3	120.3	C16—C15—H15	119.8
C5—C4—C3	122.1 (3)	C11—C16—C15	118.8 (3)
C5—C4—Cl1	119.1 (2)	C11—C16—H16	120.6
C3—C4—Cl1	118.8 (2)	C15—C16—H16	120.6
C4—C5—C6	118.8 (3)	C17—N3—C18	121.6 (2)
C4—C5—H5	120.6	C17—N3—C19	121.2 (2)
C6—C5—H5	120.6	C18—N3—C19	117.2 (3)
C5—C6—C1	119.5 (2)	O3—C17—N3	126.3 (3)
C5—C6—C7	124.8 (2)	O3—C17—H17	116.8
C1—C6—C7	115.7 (2)	N3—C17—H17	116.8
N1—C7—C8	112.2 (2)	N3—C18—H18A	109.5
N1—C7—C6	127.8 (2)	N3—C18—H18B	109.5
C8—C7—C6	120.0 (2)	H18A—C18—H18B	109.5
C10—C8—C7	105.3 (2)	N3—C18—H18C	109.5
C10—C8—C9	131.7 (3)	H18A—C18—H18C	109.5
C7—C8—C9	123.0 (2)	H18B—C18—H18C	109.5
O2—C9—O1	117.0 (2)	N3—C19—H19A	109.5
O2—C9—C8	128.1 (2)	N3—C19—H19B	109.5
O1—C9—C8	114.8 (2)	H19A—C19—H19B	109.5
N2—C10—C8	105.8 (2)	N3—C19—H19C	109.5
N2—C10—H10	127.1	H19A—C19—H19C	109.5
C8—C10—H10	127.1	H19B—C19—H19C	109.5
C12—C11—C16	121.5 (2)		

### Hydrogen-bond geometry (Å, °)

**Table:**

*D*—H···*A*	*D*—H	H···*A*	*D*···*A*	*D*—H···*A*
C10—H10···O3	0.95	2.19	3.122 (3)	167
C16—H16···O3	0.95	2.49	3.415 (3)	164
